# The neglected majority: Inverse relationship between prevalence and global research activity of 15 dermatological diseases

**DOI:** 10.1111/ddg.15885

**Published:** 2025-10-16

**Authors:** Oliver Brandt, Erere Otrofanowei, Ncoza Dlova, Simon M. Mueller

**Affiliations:** ^1^ Department of Dermatology University Hospital Basel University of Basel, Switzerland; ^2^ Department of Medicine College of Medicine University of Lagos Lagos Nigeria; ^3^ Department of Dermatology Nelson R. Mandela School of Medicine University of KwaZulu‐Natal Durban South Africa

**Keywords:** Clinical trials, dermatology research, global health, health inequality, neglected tropical diseases

Dear Editors,

Diseases of the skin and its appendages are common health problems affecting almost one‐third of the world's population.^1^ Since 2007, the World Health Organization (WHO) has engaged in the “control, elimination, and eradication of neglected tropical diseases” (NTDs), which are high burdens in impoverished areas, yet have been largely overlooked by researchers in high‐income countries and the pharmaceutical industry. Skin NTDs include Buruli ulcer, cutaneous leishmaniasis, post‐kala‐azar dermal leishmaniasis, leprosy, lymphatic filariasis and related disorders, mycetoma, deep fungal infections, onchocerciasis, scabies, and yaws.^2^ These diseases often bring stigma and discrimination to those affected and may cause lasting disabilities, potentially affecting patient`s mental well‐being, job opportunities and income, as well as their overall quality of life.^3^

Research, particularly clinical trials, is the key to drive medical progress. Ideally, global research activity should reflect the leading problems proportionally. However, it is unknown if and how the past and near future research activity correlates with the globally leading dermatological problems. To explore this issue, we analyzed the relationship between their prevalence and global research activity.

Based on categories classified in the Global Burden of Disease (GBD) study 2019 and related publications,^4^, ^5^ we included the 13 listed dermatological diseases plus melanoma and non‐melanoma skin cancer in this analysis. Age‐standardized prevalence rates were used that allowed comparison of rates across different geographical regions or time periods. These rates were obtained from the GBD online database (https://vizhub.healthdata.org/gbd‐results/), which to our knowledge represents the most comprehensive global repository of epidemiological data. The 15 diagnoses, listed with descending age‐standardized prevalence rates, are: 1. Fungal skin diseases, 2. Acne vulgaris, 3. Scabies, 4. Atopic dermatitis, 5. Viral skin diseases, 6. Contact dermatitis, 7. Pruritus, 8. Urticaria, 9. Bacterial skin diseases, 10. Psoriasis, 11. Seborrheic dermatitis, 12. Alopecia areata, 13. Non‐melanoma skin cancer, 14. Melanoma, and 15. Decubitus ulcer.

Spearman's rank correlation coefficient was used to calculate the correlation of the age‐standardized prevalence rate with global past and future research activity.

For each diagnosis, we have determined the number of clinical trials published in PubMed over the past 20 years (past research activity) as well as the number of ongoing clinical trials registered at clinicaltrials.gov (future research activity) as of April 21, 2023.

As shown in Figure 1, there is an evident, albeit not statistically significant inverse correlation in both graphs. With respect to “past research activity” (Figure 1a), an inverse trend is evident between the age‐standardized prevalence rate and the number of clinical trials published (Spearman's rho = –0.15, p = 0.58). Between 2003 and 2023, the most clinical trials were published on melanoma, pruritus, and psoriasis; while the least studies were published on alopecia areata, seborrheic dermatitis, and scabies (Figure 1a).

**FIGURE 1:**
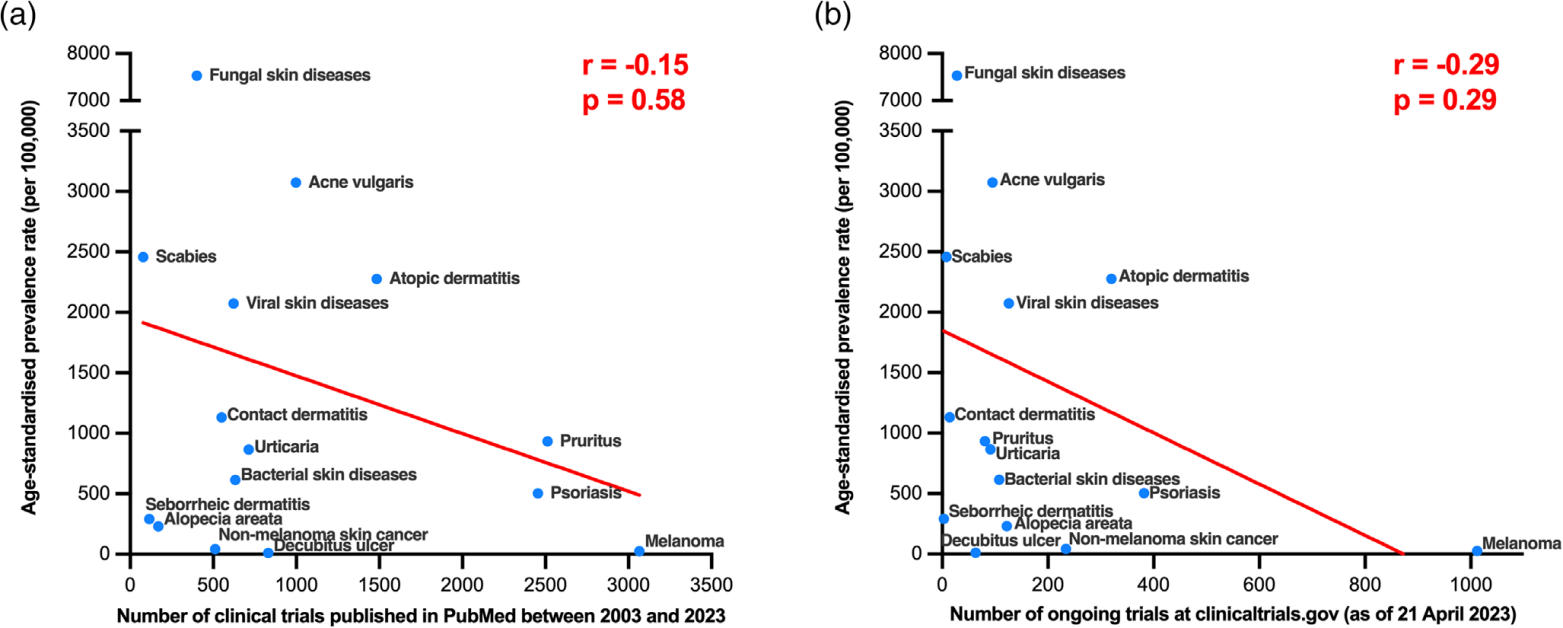
Correlation of the age‐standardized prevalence rate with global past and future research activity of 15 dermatological diseases. (a) Correlation between the age‐standardized prevalence rate and the number of clinical trials listed in PubMed between 2003 and 2023. (b) Correlation between the age‐standardized prevalence rate and the number of ongoing clinical trials on clinicaltrials.gov as of April 21, 2023.

In the context of “future research activity”, the inverse relationship trend is even more obvious (Figure 1b; Spearman's rho = –0.29, p = 0.29). The number of ongoing clinical trials on melanoma (n = 1012) far exceeds that of other dermatological diseases and is three times that of psoriasis (n = 382), which ranks second. In contrast, seborrheic dermatitis (n = 3) and scabies (n = 8) have the least ongoing clinical trials. Notably, despite being the most prevalent dermatological diseases worldwide and affecting approximately one billion people,^6^ fungal skin diseases only rank 12th in both the number of published clinical trials in PubMed and ongoing clinical trials, among all 15 diagnoses.

Since the *Global Forum for Health Research* coined the term 10/90 gap more than 25 years ago to denounce the fact that less than 10% of global healthcare research expenditure is directed at health problems that account for more than 90% of the global disease burden, it appears that no fundamental changes have occurred. Our analysis revealed that some less common dermatological diseases are receiving disproportionately high attention. This is exemplified by the fact that the highest past and future research activity was observed on melanoma, which, in 2020, accounted for an estimated 325,000 new cases globally (translating into an incidence rate of 0.004% in a world population of 8.1 billion) and primarily affects the fair‐skinned population (Fitzpatrick skin types I–II), who have a 30‐fold higher risk compared to individuals with darker skin tones.^7^

Furthermore, while melanoma was responsible for approximately 57,000 deaths worldwide in 2020,^8^ the mortality rate for scabies, an infestation which affects some 200 million people and is often still regarded a harmless skin disease, is far exceeded. In severe infestations, such as “crusted scabies”, and in populations where scabies is endemic, bacterial superinfections develop into severe skin, soft tissue, and/or invasive infections in 41 to 93% of individuals and are fatal in up to 16%.^9^, ^10^

Yet it is obvious that most skin NTDs, likewise e.g. cutaneous leishmaniasis, are usually neither life‐threatening nor directly lethal. However, many of these diseases are associated with a high overall burden on those affected, as reflected in the *Disability‐Adjusted Life Year (DALY) values*, a figure indicating the number of healthy life years per 100,000 individuals that are lost due to illness or premature death.

With a global DALY of 67, scabies again presents a striking example, exceeding those of melanoma and psoriasis, which account for 21 and 47, respectively (GBD online database; https://vizhub.healthdata.org/gbd‐results/). However, incidence, prevalence, and DALYs of diseases vary considerably across world regions, and the majority of fair‐skinned people lives in member countries of the Organisation for Economic Co‐operation and Development (OECD), where most research is conducted, and newly developed drugs are marketed.

Limitations of our study are: *(1)* only English‐language articles were included; *(2)* other large databases, e.g., Chinese Clinical Trial Registry (www.chictr.org.cn), were not considered; *(3)* DALY values were not given for all dermatoses mentioned.

In conclusion, our findings indicate that global research activity is disproportionately focused on a privileged minority in well‐resourced countries with predominantly fair‐skinned populations and on dermatological conditions prevalent among them. To address this imbalance, strong advocacy for all patients, along with targeted and coordinated efforts by the scientific community, health organizations, foundations, and international governments, is required. In addition, the pharmaceutical industry in particular, given its central role in research and development, must be involved and motivated to support a broader, more inclusive research agenda that does not reduce existing priorities such as melanoma research, but rather expands them in a targeted manner by including neglected dermatoses.

## CONFLICT OF INTEREST STATEMENT

None.
